# Atypical Nuchal-Type Fibromas: A Rare Presentation of the Soft Tissue Tumor in the Bilateral Lower Extremities

**DOI:** 10.7759/cureus.83016

**Published:** 2025-04-25

**Authors:** Yasmine Kasiri, Ryan Uchimura, Bryon Thomson

**Affiliations:** 1 Department of Radiology, Riverside University Health System Medical Center, Moreno Valley, USA; 2 Radiology, Western University of Health Sciences, Pomona, USA

**Keywords:** ankle, lower extremity, nuchal-type fibroma, soft tissue, tumor

## Abstract

Nuchal-type fibromas (NTFs) are rare, benign tumors that predominantly originate in the posterior neck and often arise due to repetitive mechanical use. They are more common in males than females. Clinically, NTFs present as slow-growing, painless masses that are firm to the touch and immobile. The masses are typically not accompanied by other symptoms of erythema, warmth, or ulcerations as they are non-inflammatory in nature, with the possibility of tenderness in later stages of growth as nerves become entrapped in the tumor. Patients may present with cosmetic concerns and focal discomfort depending on the location and size of the mass. Extra-nuchal sites are uncommon, with locations reported in the literature including the upper back, shoulder, and face, with only one case reported where an NTF was found unilaterally on the ankle. We present a rare case of a 48-year-old male with bilateral masses in the ankle extensor retinacula, consistent with nuchal-type fibromas determined by histopathology. To our knowledge, our patient's presentation of bilateral extra-nuchal NTFs of the lower extremity is among the first to be reported in the literature.

## Introduction

Nuchal-type fibromas (NTFs) are rare, benign tumors that arise from connective tissue, particularly affecting the posterior cervical region in the form of a palpable mass. NTFs commonly appear in the age range of 40-50, with a 4:1 predominance in males and a strong association with diabetes mellitus, with about 44% of recorded cases affecting patients who were diagnosed with diabetes [1]. Clinically, NTFs present as a slow-growing, painless mass of the dorsocervical region that is firm to the touch and immobile [2-6]. The masses are typically not accompanied by other symptoms of erythema, warmth, or ulcerations as they are non-inflammatory in nature, with the possibility of tenderness in later stages of growth as nerves become entrapped in the tumor. Patients may present with cosmetic concerns and focal discomfort depending on the location and size of the mass. Extra-nuchal sites are uncommon, with locations reported in the literature including the upper back, shoulder, and face, with only one case reported where an NTF was found unilaterally on the ankle [2-4, 7]. While the etiology remains unclear, these pseudotumors may arise due to repetitive trauma, with various cases describing NTFs in male weight lifters [5-6, 8]. Histopathological features of NTFs describe ill-defined, thick bundles of hypocellular collagen that trap nearby structures such as adipose tissue and nerves [1, 4-5, 9, 10]. Ultrasound and X-ray are often the initial imaging modalities used to characterize NTFs, demonstrating a nonspecific hypoechoic focus with irregular borders or a radiopaque soft tissue density, respectively [4]. MRI provides the best visualization of the mass with typical imaging characteristics of low signal intensity on T1 and mixed signal intensity on T2 with avid enhancement [1, 4].

## Case presentation

**Figure 1:**
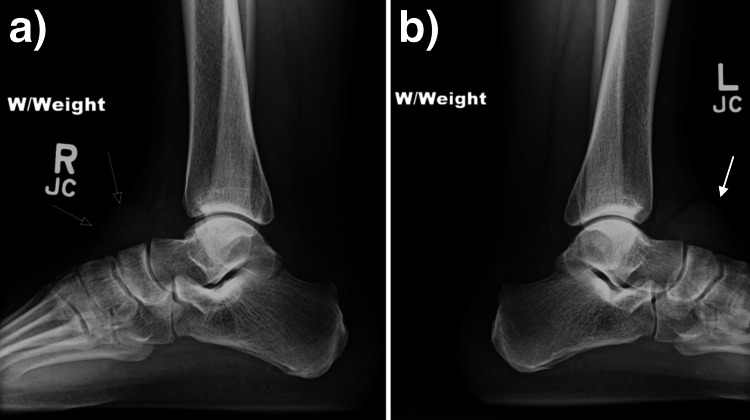
Weight-bearing X-rays of the right (a) and left (b) ankles demonstrating rounded soft-tissue densities within the bilateral dorsolateral soft tissues (arrows).

**Figure 2:**
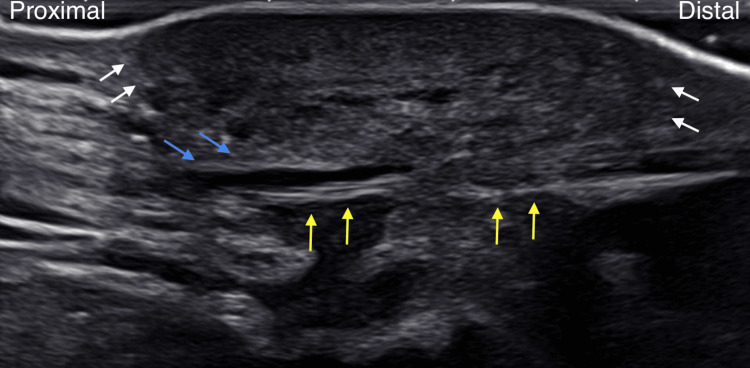
Long-axis ultrasound of the left anterolateral ankle showing a heterogenous hypoechoic mass with irregular margins (white arrows) anterior to the extensor retinaculum (blue arrows pointing to the superior band of the extensor retinaculum and yellow arrows pointing to inferior band).

**Figure 3:**
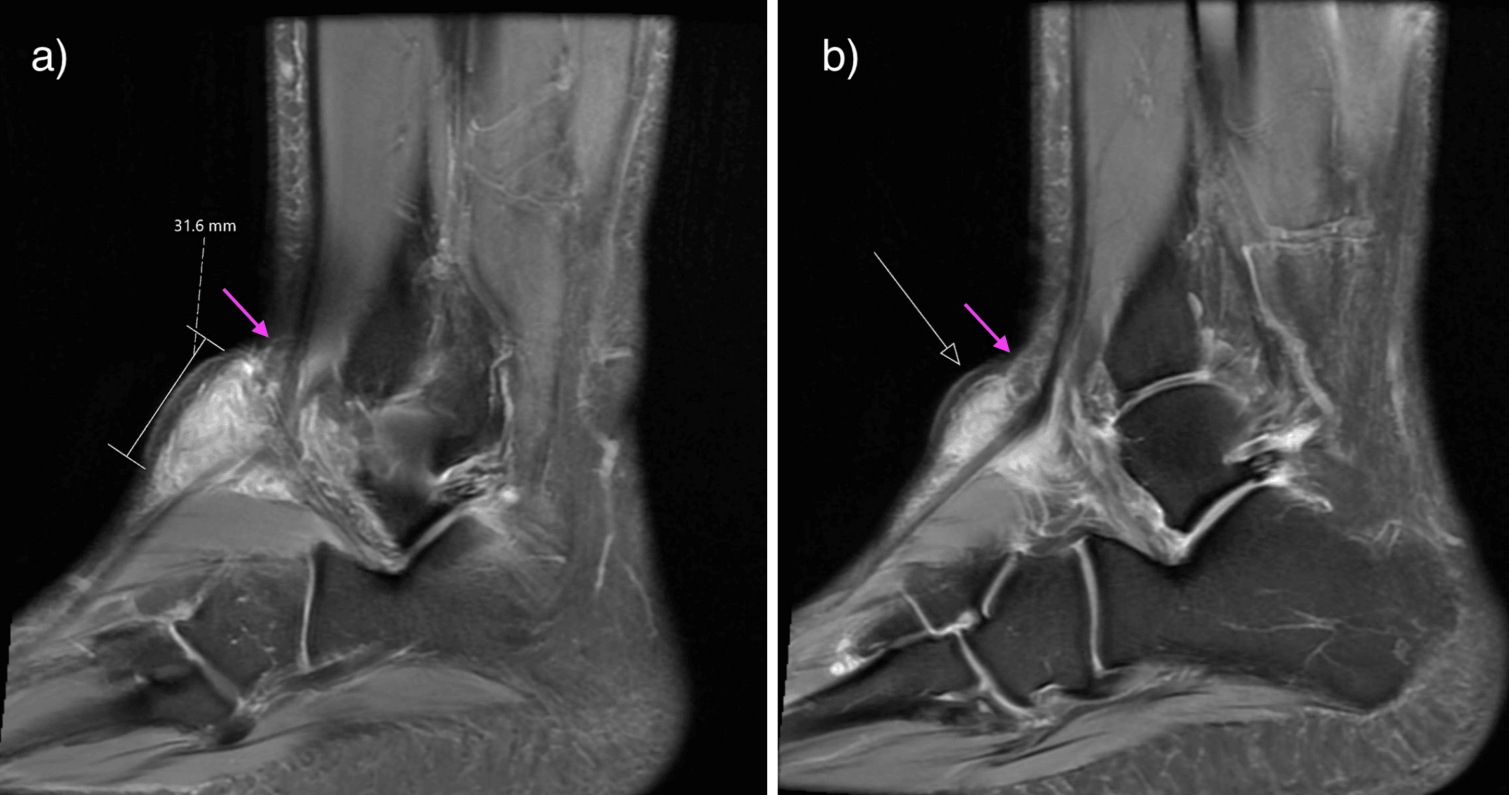
Sagittal Proton Density fat-saturated images of the right (a) and left (b) ankles, displaying hyperintense masses within the dorsolateral soft tissues with proximal fat locules (pink arrows).

Our patient is a 48-year-old man who presented with bilateral, firm, mobile masses on the anterolateral ankles, seeking consultation for surgical removal. At the time of the presentation, the patient reported that he had had the masses for 10 years and that they had been tender for all 10 years, with tenderness worsening within the past year. The masses were firm and did not adhere to the skin. The patient denied recent changes in the size of the masses. He also denied any trauma to the areas, but reported that he worked as a landscaper and wore boots that came into contact with the masses daily. Weight-bearing ankle X-rays demonstrated bilateral soft-tissue masses on the dorsal aspect of the ankles (Fig. 1) Ultrasound findings were generally unclear, describing skin thickening with ill-defined heterogeneous hypoechogenicity in the subcutaneous tissues on the anterolateral aspects of both ankles (Fig. 2). MRI with and without administration of contrast revealed ellipsoid masslike areas bilaterally, exhibiting predominantly low T1 signal with some locules of fat at the proximal and distal extents, heterogeneously hyperintense T2 and PD signal, and subtle areas of internal enhancement on postcontrast imaging (Fig. 3).

**Figure 4:**
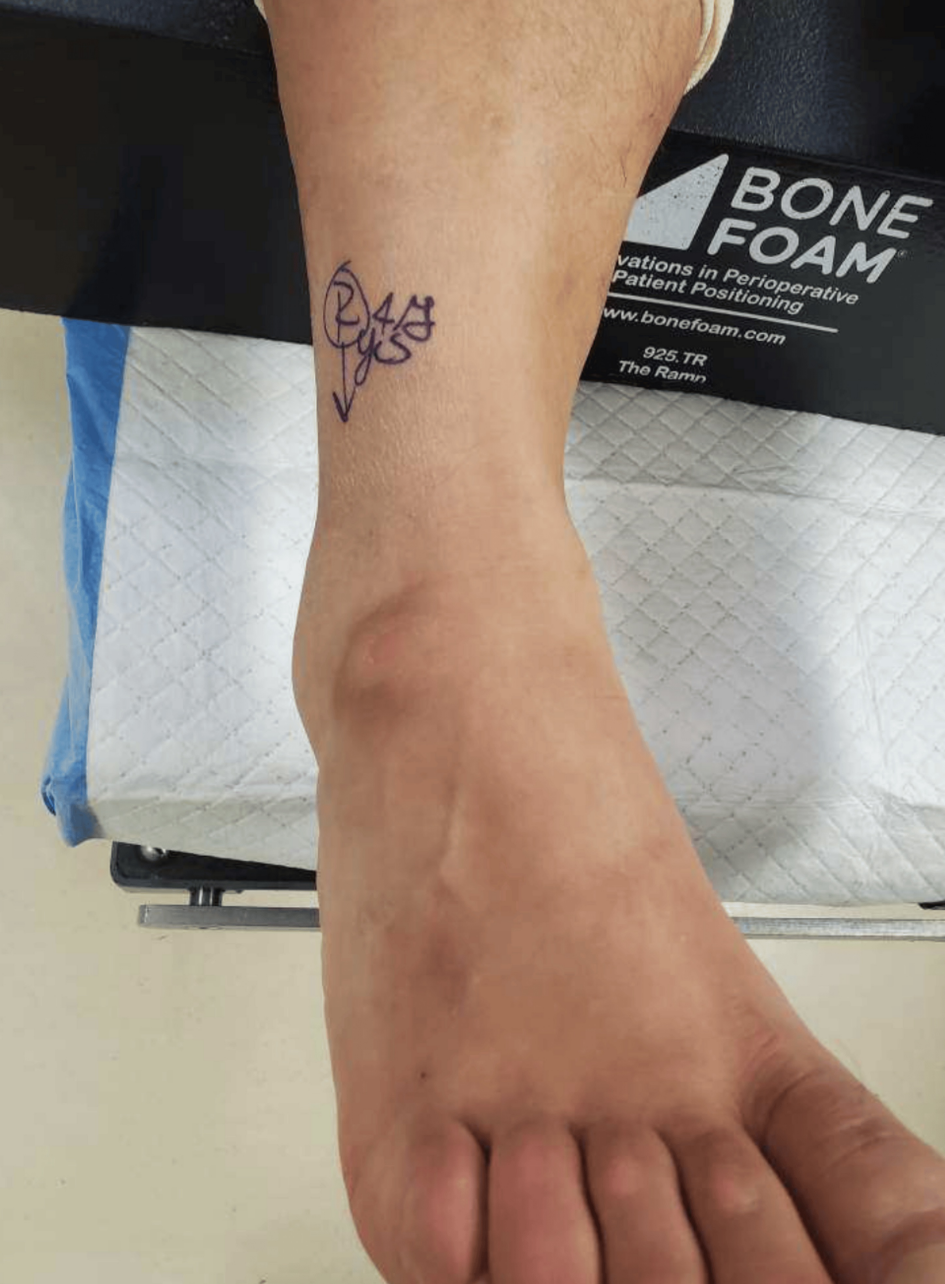
Image of dorsolateral foot mass on right lower extremity, prior to surgical removal.

**Figure 5:**
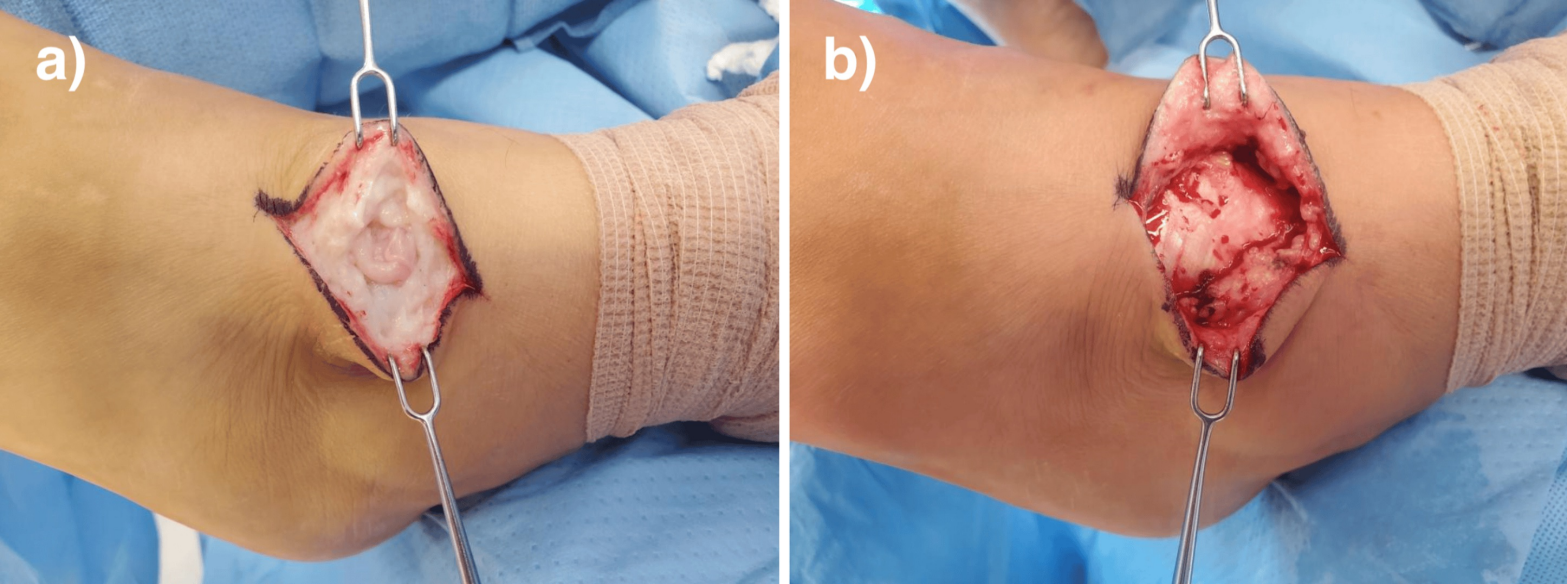
Intraoperative images of the left (a) and right (b) foot masses.

**Figure 6:**
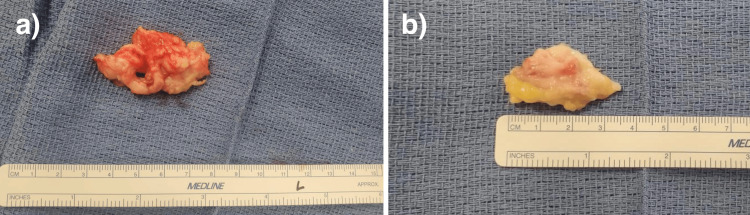
Gross pathology after excision of left (a) and right (b) foot masses, respectively, measuring 4.7 x 3.0 x 1.1 cm and 3.2 x 1.8 x 1.0 cm.

**Figure 7:**
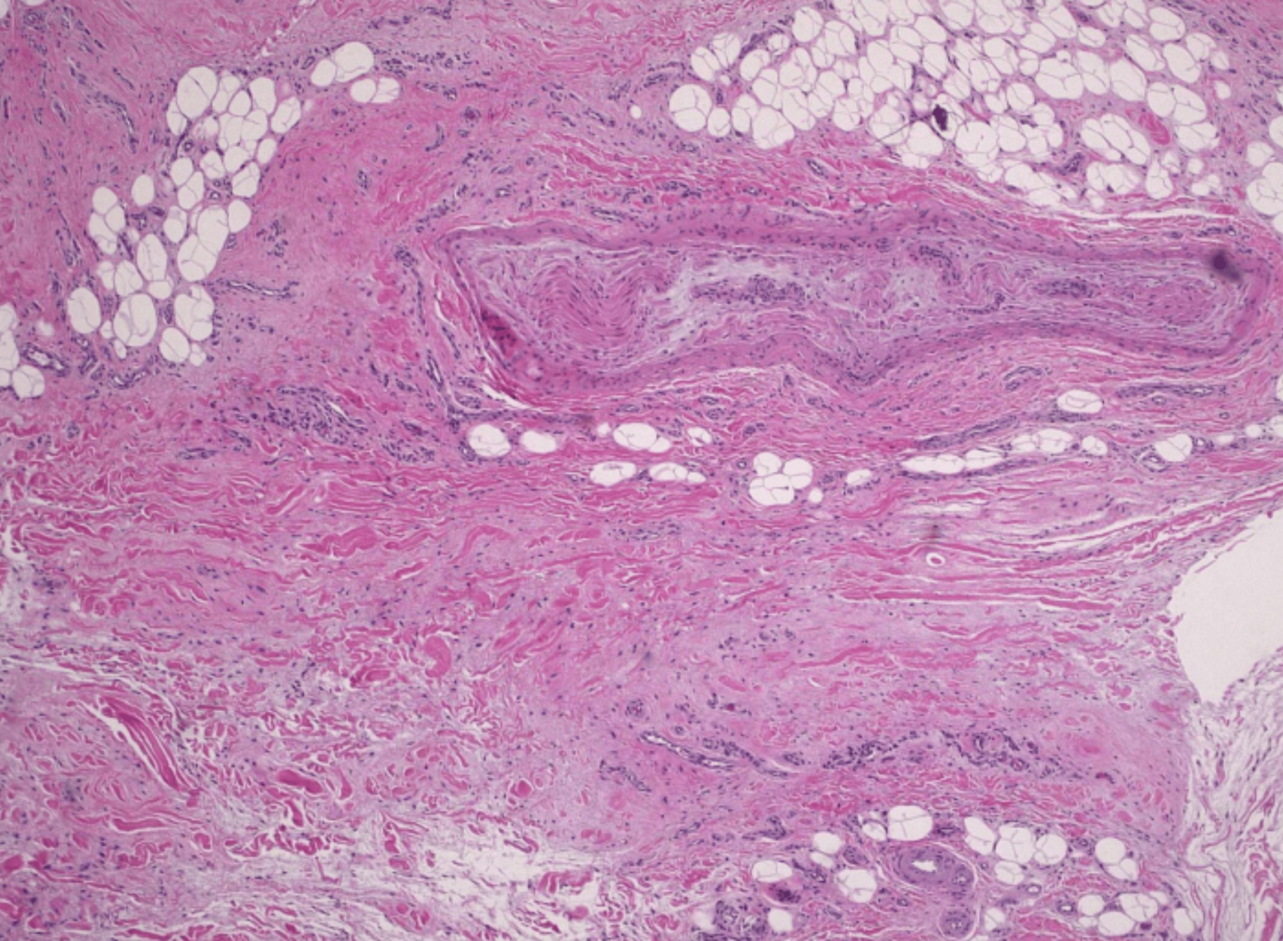
Hematoxylin and eosin stain at x10 magnification of excised mass, demonstrating stroma with coarse bundles of acellular collagen surrounding nerves.

Bilateral surgical dissection was performed through soft tissue with retraction of all neurovascular structures (Fig. 4, 5). Operative findings described bilateral fibrous, well-defined masses that remained superficial to the inferior extensor retinaculum. Gross pathological findings described 3.2 x 1.8 x 1.0 cm and 4.7 x 3.0 x 1.1 cm fragments of yellow-to-white rubbery tissue with white, smooth homogenous to fatty cut surface (Fig. 6). These were then histopathologically characterized as benign nuchal-type fibromas, showing stroma with coarse acellular collagen bundles focally surrounding nerves (Fig. 7).

## Discussion

Our patient presented with bilateral NTFs determined by histology. The etiology of NTFs is largely unknown and has associations with diabetes mellitus, Gardner syndrome, and repetitive physical trauma [4-6, 11-12]. Our patient’s occupation as a landscaper, where he self-reported spending long hours wearing boots that rubbed against his ankles and frequently kneeling, may support this repetitive trauma hypothesis as a contributing factor to NTF development [8-9, 11]. While NTFs are not inflammatory in nature, the chronic mechanical stress and friction in his case could have triggered a reactive fibrotic process, leading to the formation of these bilateral masses. This aligns with previous reports associating NTFs with repetitive mechanical use in physically active individuals. Despite the absence of overt inflammatory changes in such individuals, the role of chronic low-grade trauma in NTF pathogenesis warrants further investigation.

Overall incidence of NTFs is incredibly low, with about only 100 cases reported in the literature, and about 70% of cases presenting as posterior neck masses [1, 7]. These rare, benign soft-tissue tumors are described histopathologically as thickened, disorganized bundles of collagen that entrap surrounding adipose, nerves, and vessels [1, 4, 6-7, 10]. The histology findings of our case corroborate this, where bundles of collagen haphazardly surrounded nerves were seen; this may explain why our patient began to experience tenderness in the masses as they grew over time [1, 10]. While NTFs are benign with a good prognosis, the recommended treatment is complete excision of the masses with monitoring, as the recurrence rate is reported to be between 13-20% [1]. Malignant potential is exceedingly low, with no reported cases of malignant transformation of an NTF described in the current literature.

NTFs can be visualized on ultrasound and MRI, with MRI being the superior imaging study, as ultrasound is not diagnostic, where the masses are seen as hypoechoic, ill-defined lesions. Typical MRI findings include T1 low-intensity signal, T2 low or mixed intensity signal, with enhancement [1, 8-9, 11, 13]. These findings were consistent with our case of NTFs that atypically presented bilaterally on the lower extremity.

## Conclusions

NTFs are benign soft-tissue tumors that are most commonly found in the posterior neck. This case emphasizes the importance of considering NTFs in the differential diagnosis of firm, painless soft tissue masses in extra-nuchal locations, with special considerations in patients with a history of diabetes or repetitive mechanical stress. Ultrasound and MRI imaging play an important role in assessing these lesions by providing insight on the margins and enhancement patterns of the masses, however, definitive diagnosis relies on histopathology. Complete surgical excision remains the recommended treatment with an excellent prognosis and with the risk of non-malignant recurrence.
